# Mechanical Control of Myotendinous Junction Formation and Tendon Differentiation during Development

**DOI:** 10.3389/fcell.2017.00026

**Published:** 2017-03-23

**Authors:** Mauricio Valdivia, Franco Vega-Macaya, Patricio Olguín

**Affiliations:** Program in Human Genetics, Faculty of Medicine, Institute of Biomedical Sciences, Biomedical Neurosciences Institute, University of ChileSantiago, Chile

**Keywords:** tendon cells, myotendinous junction, mechanical forces, morphogenesis, mechanoresponse

## Abstract

The development of the musculoskeletal system is a great model to study the interplay between chemical and mechanical inter-tissue signaling in cell adhesion, tissue morphogenesis and differentiation. In both vertebrates and invertebrates (e.g., *Drosophila melanogaster*) the formation of muscle-tendon interaction generates mechanical forces which are required for myotendinous junction maturation and tissue differentiation. In addition, these forces must be withstood by muscles and tendons in order to prevent detachment from each other, deformation or even losing their integrity. Extracellular matrix remodeling at the myotendinous junction is key to resist mechanical load generated by muscle contraction. Recent evidences in vertebrates indicate that mechanical forces generated during junction formation regulate chemical signaling leading to extracellular matrix remodeling, however, the mechanotransduction mechanisms associated to this response remains elusive. In addition to extracellular matrix remodeling, the ability of *Drosophila* tendon-cells to bear mechanical load depends on rearrangement of tendon cell cytoskeleton, thus studying the molecular mechanisms involved in this process is critical to understand the contribution of mechanical forces to the development of the musculoskeletal system. Here, we review recent findings regarding the role of chemical and mechanical signaling in myotendinous junction formation and tendon differentiation, and discuss molecular mechanisms of mechanotransduction that may allow tendon cells to withstand mechanical load during development of the musculoskeletal system.

## Introduction

Living cells and tissues are in a constant state of isometric tension allowing them to respond to mechanical cues (Ingber, 1997; Wang et al., 2001; Mammoto and Ingber, 2010). During embryogenesis, mechanical stress is generated within the tissue and by its interaction with external factors and/or other tissues. Shear stress generated by blood flow modulates blood vessels morphogenesis, regulates the fate acquisition of arteries and veins, and is required for the development of the hematopoietic system (le Noble et al., 2004; Adamo et al., 2009; North et al., 2009). In addition, hemodynamic forces are required for heart morphogenesis. Disturbing blood flow at either the inflow or outflow tracts of the zebrafish heart results in several defects including abnormal formation of third chamber and heart looping (Hove et al., 2003). Furthermore, mechanotransduction mechanisms and its role in development are evolutionary conserved across species. In zebrafish and *Drosophila*, mechanical cues generated during gastrulation (epiboly in zebrafish, and mesoderm invagination in flies) induce β-Catenin release from E-Cadherin based junctions, and translocation to the nucleus of mesodermal cells, where it promotes gene expression changes and cell specification (Farge, 2003; Desprat et al., 2008; Brunet et al., 2013).

The development of muscle-tendon attachment is a great model to study the role of chemical and mechanical signaling between tissues in morphogenesis and differentiation (Schweitzer et al., 2010; Subramanian and Schilling, 2015). During embryogenesis, tendon cells attach to the developing muscle through the Extracellular Matrix (ECM) forming a specialized junction called Myotendinous Junction (MTJ) (Schweitzer et al., 2010; Subramanian and Schilling, 2015). MTJ development relays mainly on the interaction of Integrins and ECM molecules secreted by tendons and muscles, although, other proteins, like Dystroglycan and Kon-tiki (Kon) also contribute to the formation of the MTJ. While Dystroglycan participates on muscle binding to the ECM, Kon controls muscle guidance and attachment to muscle attachment sites (Pérez-Moreno et al., 2014; Weitkunat et al., 2014; Maartens and Brown, 2015; Subramanian and Schilling, 2015). Strain generated by the contraction of the developing muscles contributes to MTJ maturation and muscle and tendon differentiation (Weitkunat et al., 2014; Havis et al., 2016). Here we will review recent evidences regarding the role of mechanical signaling in tendon differentiation and MTJ formation in vertebrates and *Drosophila*. Additionally, we will discuss the mechanisms of mechanoresponse that may allow tendon cells to sense and respond to mechanical load during development of the muscle-tendon interaction.

## The role of mechanical and chemical signaling in vertebrate tendon differentiation

**Figure 1:**
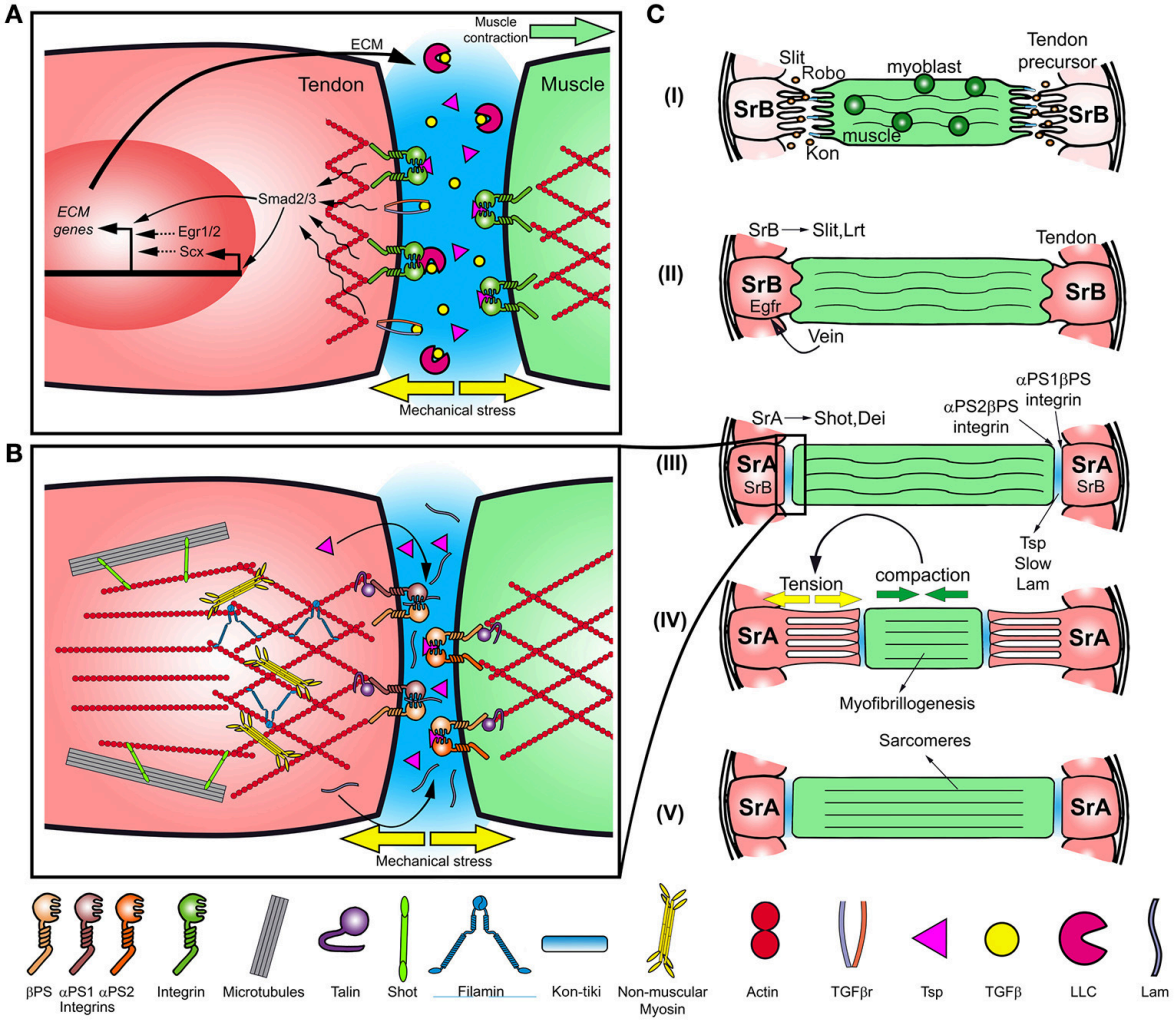
**Myotendinous junction formation in vertebrates and ***Drosophila***. (A)** Scheme of vertebrate myotendinous junction formation. Mechanical stress on the ECM may cause the release of the secreted TGFβ from the large latent complex (LLC) and activation of the receptor. In addition, TGFβ bound to LLC activates Integrin receptors. Smad2/3 along with Integrin signaling, activate Scx and Egr1/2, inducing the expression and deposition of ECM proteins. **(B)** Scheme of the myotendinous junction in *Drosophila*. In tendon cells, the link between Integrin and the actin cytoskeleton is mediated by Talin and the three-dimensional organization of the actin cytoskeleton is modulated by cross-linkers and motor proteins, such as and Filamin and Myosin. **(C)** Scheme of myotendinous system development in *Drosophila*. (I) The developing myotube migrates toward the tendon precursor cells (specified by SrB) directed by the Slit-Robo signaling and Kon-tiki, while myoblasts fuse with the myofiber. (II) After recognition tendon and myotube extensions interdigitate, in addition Vein is secreted promoting SrB expression. (III) ECM components, as Thrombospondin (Tsp) and Laminin (Lam), are secreted to the MTJ. In tendon cells, SrA is expressed and SrB expression diminishes. (IV) Myotube compacts generating mechanical stress on the system triggering myofibrillogenesis. (V) Sarcomeres are formed, and muscle elongate back toward tendon cells.

Mechanical control of tendon differentiation and remodeling has been widely studied in vertebrates (reviewed in Shwartz et al., 2013). Tendons are formed by ECM, composed principally by strong collagens fibril arrays, and a type of fibroblast termed tenocyte (Subramanian and Schilling, 2015). In response to mechanical forces, tenocytes secrete collagens and proteoglycans, modifying ECM composition and elastic properties (Chen X. et al., 2012; Li et al., 2015). These changes confer tendons with the ability to resist mechanical load generated during muscle contraction and to form functional attachments to bones (Evans and Barbenel, 1975; Kjaer and Kjær, 2004; Maeda et al., 2011; Schwartz et al., 2013; Havis et al., 2016). How force is sensed by tenocytes and transduced into a cellular response? Recent studies on the development of the MTJ shed lights into this problem. In chicks and mice, the morphogenesis of the limb MTJ is divided in two phases (Subramanian and Schilling, 2015). The first phase is independent of muscle derived signals (Pryce et al., 2009). Here, the initial expression of Scleraxis (Scx), a tendon-specific bHLH transcription factor that promotes tendon differentiation and tenocyte specification (Alberton et al., 2012; Chen L. et al., 2012; Li et al., 2015), is stimulated by Fibroblast Growth Factor (FGF) and Transforming Growth Factor-beta (TGFβ) through MAPK/ERK and SMAD2/3 signaling pathways, respectively (Schweitzer et al., 2001; Havis et al., 2014; Figure 1A). Scx mutant mice display disrupted tenocyte differentiation leading to disorganized ECM, however, tenocyte precursor cells are still specified, indicating that other genes are required for early specification (Murchison et al., 2007). During the second phase of tendon differentiation, the interaction with the developing myofiber is mandatory to maintain the expression levels of Scx and other tendon markers (Havis et al., 2016). Pharmacological inhibition of muscle contraction disturbs tendon differentiation, even in presence of FGF and TGFβ, diminishing the levels of Scx. Moreover, force exerted by muscles on tendons is required for the activation of FGF and TGFβ at the muscle-tendon interface, maintaining the expression levels of Scx, leading to tendon terminal differentiation (Maeda et al., 2011; Havis et al., 2016).

TGFβ-ligands are secreted bound to TGFβ-binding proteins which form a complex with the large latency complex (LLC) in the ECM, capturing TGFβ and precluding its binding to TGFβ-receptors (Wipff et al., 2007; Maeda et al., 2011; Figure 1A). Shearing forces generated during muscle contraction may stimulate TGFβ release from the LLC through its degradation by proteases, allowing its binding to the receptor (Figure 1A). Moreover, it may promote the activation of Integrin signaling through the binding of the RGD motifs present on the latency TGFβ binding proteins associated to LLC (Munger and Sheppard, 2011; Subramanian and Schilling, 2015; Figure 1A). TGFβ signaling maintains Scx expression under normal muscular-load regime in mice (Maeda et al., 2010, 2011) and in response to mechanical stress promotes expression of Integrins (Popov et al., 2015). Thus, different mechanotransduction mechanisms appear to function at the ECM levels, activating either TGFβ or Integrin signaling. In vertebrates, recent evidence have shown that mechanical forces appears to be required for muscle development. Mechanical force driven by muscle contraction is necessary to maintain the pool of muscle progenitors during chick fetal myogenesis (de Lima et al., 2016), and *in vitro* studies suggest that strain drives mesenchymal stem cells differentiation into myoblasts (Lisio et al., 2014; Lemke and Schnorrer, in press).

## The role of mechanical signaling in *Drosophila* myotendinous junction formation and tendon differentiation

In contrast to vertebrates, *Drosophila* displays an exoskeleton instead of an internal skeleton and its connection with muscles relays on epithelial cells of ectodermal origin called tendon cells, which are analogs to vertebrate tendons (Fernandes et al., 1996; Figure 1B). Similar to vertebrates, signals emanated from tendon cells are required for MTJ formation, both during embryogenesis and metamorphosis (Costello and Wyman, 1986; Fernandes et al., 1991; Wayburn and Volk, 2009; Ordan et al., 2015). In order to resist mechanical load, tendon cells modify their elastic properties deploying an array of polarized microtubules and actin filaments that stretch along their apical-basal axis, from the exoskeleton attachment site to the MTJ (Subramanian et al., 2003; Alves-Silva et al., 2008).

The development of the interaction between the Indirect Flight Muscles (IFMs) and the tendon cells of the dorsal thorax (notum) is an interesting model to study the role of mechanical signaling in tissue morphogenesis and cell differentiation (Olguín et al., 2011; Weitkunat et al., 2014). The notum develops from a monolayer epithelium, from which a subset of epithelial cells differentiates as analogs to vertebrate tendons, serving as bridges between the flight muscles and the exoskeleton (Fernandes et al., 1991; Weitkunat et al., 2014). At early stages of tendon differentiation, tendon precursors are specified by the activity of the isoform B of the Stripe transcription factor (SrB), which is required and sufficient to specify tendon cells (Volk and VijayRaghavan, 1994; Frommer et al., 1996; Becker et al., 1997; Figure 1C). The *stripe* homologous in vertebrates, Egr1 and Egr2, are required for tendon terminal differentiation, specifically to promote the expression of ECM proteins (Frommer et al., 1996; Lejard et al., 2011; Guerquin et al., 2013), however, as Scx, they are not strictly required for tendon specification (Lejard et al., 2011; Guerquin et al., 2013). Once specified, embryonic tendon cells provide initial attracting cues to the myotube and secrete Slit, a ligand that binds Robo receptor, which is expressed at the tips of myotubes (Figure 1C; Kramer et al., 2001; Ordan et al., 2015). Whether Slit acts as a chemoattractant in this context, remains to be elucidated. During this first stage of myotendinous system development, myotubes extend bipolar extensions that migrate toward their tendon targets, conversely, tendon cells extend processes that interact with the myotube extension tips (Figure 1C; Vega-Macaya et al., 2016). Muscle migration requires the accumulation of Kon, a single pass transmembrane protein, on the muscle leading ends (Figure 1C; Estrada et al., 2007; Schnorrer et al., 2007). Loss of function of Kon in the ventral longitudinal muscles causes abnormal projection of filopodia, altering the myotube migration pattern (Schnorrer et al., 2007). Following, in a second stage, myotubes secrete Vein, a short range signaling molecule that binds to the epidermal growth factor receptor (EGFR) expressed in tendon cells, promoting SrB expression (Yarnitzky et al., 1997; Figure 1C). High levels of SrB induce Slit secretion and Leucine Rich repeat Transmembrane protein (LRT) expression, which bind to Robo and are both required for muscle migration arrest (Figure 1C; Wayburn and Volk, 2009; Ordan and Volk, 2015, 2016). Slit acts as a short range repellent signal that arrests muscle migration. This mechanism depends on Slit cleavage by Amontillado, a Pheromone Convertase 2 homolog, sequestering Slit on the tendon cell membrane, stopping muscle migration (Ordan et al., 2015; Ordan and Volk, 2016). In a third stage, the MTJ starts forming mainly through the association of Integrin with ECM proteins secreted by tendon and myotube (Chanana et al., 2007; Subramanian et al., 2007; Gilsohn and Volk, 2010; Figures 1B,C). The muscle-specific αPS2βPS Integrin binds to Thrombospondin (Tsp) and its regulator Slow, conversely, Laminin (Lam) associates with the tendon-specific αPS1βPS Integrin (Gotwals et al., 1994; Martin et al., 1999). The induction of SrA isoform and the decrease of SrB expression levels is essential to promote the expression of tendon specific differentiation genes such as *Delilah* (Dei), a transcription factor that promotes βPS expression, and *shortstop/kakapo* (Shot), a plakin that connects the actin cytoskeleton to microtubules, regulating the elastic properties of tendon cells (Subramanian et al., 2003; Schweitzer et al., 2010). Thus, during this stage EGFR and Integrin signaling promotes junction formation and terminal differentiation of tendon cells.

During metamorphosis, developing tendons and muscles express the same combinations of Integrin subunits and secrete extracellular matrix components such as Tsp, forming stable hemiadherent junctions (Subramanian et al., 2007; Gilsohn and Volk, 2010; Weitkunat et al., 2014). Following, IFMs compaction, driven by Myosin Heavy Chain (MHC) motor activity, generates mechanical strain at the MTJ (Weitkunat et al., 2014; Figure 1C). In addition, the overlying notum epithelium migrates toward anterior through a still unknown mechanism, which may contribute to the mechanical strain generated between these tissues (Bosveld et al., 2012). Recently, it has been shown that mechanical strain at the MTJ is required for myofibrillogenesis, indicating that mechanical signaling is also required for muscle morphogenesis (Weitkunat et al., 2014). In response to muscle compaction, tendon extensions attached to the myotube elongate (Weitkunat et al., 2014; Figure 1C). During this process, MTJ must be able to withstand mechanical load, and tendon cells might regulate its elastic properties in order to maintain its integrity and shape.

## Membrane mechanoreceptors and mechanical signaling at the myotendinous system

**Figure 2:**
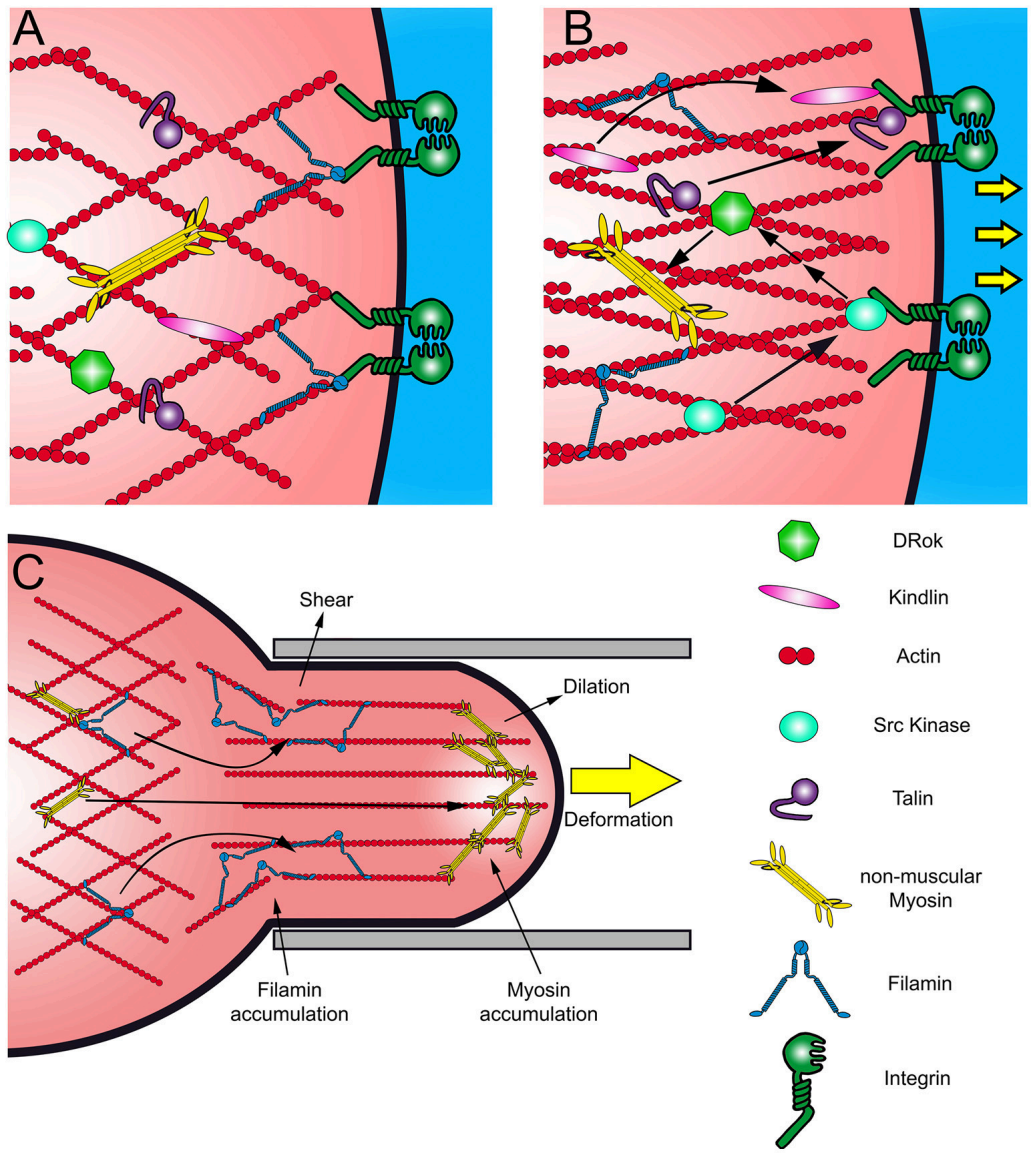
**(A,B)** Scheme of canonical cell response to mechanical stimuli. Mechanical stress results in Integrin activation and the recruitment of Kindlin and Talin, rearranging the actin network. The recruitment of Src kinase activates several pathways in response to the stress, like the Rho-ROCK pathway. **(C)** Scheme of a cell aspirated by micropipette and the redistribution of Myosin II and Filamin. These proteins accumulate as an immediate response to different types of mechanical stimuli. Filamins accumulate in response to shear stress and Myosin II in response to dilation stress.

At focal adhesions, the Integrin signaling pathway might be triggered in response to deformation or changes in the rigidity of the ECM (outside-in activation) (Takagi et al., 2003; Campbell and Humphries, 2011). In absence of external forces, Integrins remain in a restings state, associated with Filamin (Figure 2A). Mechanical stimuli may cause the opening of the extracellular domains of the Integrin heterodimer, which is transmitted to its cytoplasmic portion where it could recruit the actin binding protein Talin, although it is not the most characterized mechanism of Integrin signaling (Nieves et al., 2010; Figure 2B). The activation of Integrins also results in the recruitment of several other proteins, like Src kinases, promoting cell proliferation and migration (Arias-Salgado et al., 2003). Importantly, Src activates Rho signaling pathway, which through Rho-kinase (ROCK) induces the phosphorylation of the myosin regulatory light chain (MRLC) and the contraction of the acto-myosin network, building up tension at the focal adhesions (Arthur et al., 2000; Arias-Salgado et al., 2003). The Integrin signaling cascade may be activated also by an inside-out mechanism (Otoole et al., 1994; Vinogradova et al., 2002). There is evidence that certain proteins, like Talin, are able to respond to mechanical deformation (Lee et al., 2007; del Rio et al., 2009). *In vitro* studies have shown that Talin has cryptic vinculin interacting domains that are exposed by deformation (Lee et al., 2007; del Rio et al., 2009). Stretching of the actin cytoskeleton may be directly transmitted to Talin, releasing its Vinculin binding site, triggering the recruitment of Talin and Vinculin toward Integrins, promoting adhesion.

Integrin-ECM interaction plays an important role in the formation of the vertebrate and *Drosophila* MTJ (Brown, 2000; Figure 1A). *Drosophila* mutant embryos for either βPS Integrin or *tsp* show detachment of developing muscle fibers from tendons, due to the loss of the αPS2βPS-Tsp interaction (Chanana et al., 2007; Subramanian et al., 2007; Figure 1B). Moreover, Talin mutants display similar defects suggesting that Talin-related signaling is required for functional MTJ formation (Brown et al., 2002).

Similar to Focal adhesion, activation of Rho signaling downstream of Integrins appears to be indispensable for MTJ formation (reviewed in Geiger and Bershadsky, 2002). We have recently shown that *Drosophila* Rho-kinase (DRok) loss of function in tendon cells results in diminished phosphorylation of MRLC and abnormal βPS localization and Tsp accumulation at the MTJ, suggesting that DRok could be part of the inside-out mechanism of Integrin activation (Vega-Macaya et al., 2016). Interestingly, ROCK activity appears to be required for stretch-induced tenocyte differentiation from human Mesenchymal Stem Cells (hMSCs) (Xu et al., 2012). Stretching of hMSCs elicited enhanced expression of Scx, Collagen I and II, among other tendon specific genes. The addition of a ROCK inhibitor results in an attenuated expression of these genes (Xu et al., 2012). Whether DRok activity contributes to tendon cell differentiation in response to mechanical forces, through regulation of gene expression in *Drosophila* remains to be explored. In addition to its role in MTJ maturation, DRok regulates the orientation of tendon extensions toward IFMs during recognition stage, enabling the correct attachment to the muscle fibers (Vega-Macaya et al., 2016; Figure 1C). *DRok* mutant tendon cells display miss-oriented tendon extensions, resulting in irregular attachments to the muscle fiber. Tendon extensions appears to be unable to resist the pulling forces generated by IFMs compaction, resulting in muscle detachment and death (Vega-Macaya et al., 2016). How DRok regulates tendon recognition of the myotube ends remains to be elucidated.

In conclusion, the membrane mechanoreceptor model explains how forces are sensed and transduced at the MTJ, but how tension exerted by muscle compaction is withstood by the whole tendon cell is still unclear.

## Actin crosslinkers as intracellular mechanosensors and regulators of the actin network

*In vitro* and *in vivo* experiments show that mechanical perturbation of cell shape causes a redistribution of actin crosslinkers and a rearrangement of the actin network (Gardel et al., 2004; Chaudhuri et al., 2007; Luo et al., 2013). Studies in *Drosophila* epithelial cells, *Dyctiostelium discoideum* and mammalian cells have demonstrated that mechanical deformation of the plasma membrane results in accumulation of crosslinking and motor proteins such as Filamin and myosin, respectively, to the perturbation site in distinctive ways (Fernandez-Gonzalez et al., 2009; Luo et al., 2013; Schiffhauer et al., 2016; Figure 2C). Myosin is recruited to regions under dilation stress, counteracting cell deformation by contraction of the acto-myosin filaments (Figure 2C). On the other hand, Filamin is recruited to sites subjected to shear stress (Luo et al., 2013; Schiffhauer et al., 2016; Figure 2C).

In contrast to Myosin, Filamin does not act as a contractile unit; instead, it enhances elasticity of the actin network to allow cell shape adaptation and remodeling (Luo et al., 2013; Schiffhauer et al., 2016). Filamin is a large actin-binding protein that works as a dimer (Noegel et al., 2004). Each Filamin monomer binds to one actin filament forming orthogonal and elastic actin networks by dimerization via their C-terminal immunoglobulin-like domains (Tseng et al., 2004; Pudas et al., 2005; Figures 2A–C). Both, Jitterbug, one of the two Filamins present in *Drosophila*, and non-muscle Myosin II (MyoII) are required to maintain the shape and polarity of tendon cells and partially co-distribute with actin filaments and Shot (Olguín et al., 2011). Interestingly, Shot loss of function display similar epithelial deformation phenotypes to Jbug (Olguín et al., 2011), suggesting that both microtubule and actin arrays that stretched along the apical-basal axis of tendon cells are required to withstand mechanical load.

At the signal-transduction level, Filamin acts as a scaffold for other actin regulatory proteins (Popowicz et al., 2006). In monocytes, Filamin recruits the small GTPases of the Rho family, their effectors and regulators (Leung et al., 2010). In migrating mammalian cells, Filamin recruits ROCK (Ueda et al., 2003), which may promote acto-myosin network contraction and stabilization by activation of the myosin regulatory light chain, α-Adducin and LIMK (Maekawa et al., 1999; Zhang et al., 2003). During cell migration, Filamin also interacts with the Integrin beta subunit, keeping it in a resting state, preventing focal adhesion formation (Liu et al., 2015; Figure 2A). A proposed mechanism is that after Integrin interaction with a stiffer ECM, Filamin dissociates from Integrin cytoplasmic domain leading to Talin and Vinculin recruitment in its place, reinforcing adhesion (Nieves et al., 2010; Figure 2B).

Based on these evidences, Filamin could play a dual role in tendon cell mechanoresponse during MTJ formation: as a molecular scaffold for actin regulators at the MTJ, and as regulator of tendon cell elastic properties at specific cellular regions. Moreover, Filamin redistribution may regulate its role as a scaffold at the MTJ.

## Concluding remarks

The ability of cells and tissues to respond to mechanical stress during development is crucial to shape organs and the whole individual. The combination of molecular tools that allows to measure in developing animals, mechanical stress across developmental fields, dynamic signaling pathway activity and cytoskeleton organization will be key to unveil the interplay between mechanical and chemical signaling during embryogenesis, including the formation of the musculoskeletal system.

## Author contribution

MV and FV contributed equally to this work. MV wrote sections of the manuscript, then contributed to its editing and final formatting. FV made Figures 1, 2. PO contributed to writing, editing materials written by MV and FV, and final integration of the various sections.

## Funding

This work was supported by DRiDANS, PIA ACT-1401 and Biomedical Neuroscience Institute, Iniciativa Científica Milenio, ICM P09015F.

### Conflict of interest statement

The authors declare that the research was conducted in the absence of any commercial or financial relationships that could be construed as a potential conflict of interest.
